# Proceedings of the Meeting of the Michigan State Dental Association

**Published:** 1896-10

**Authors:** 


### THE DENTAL REGISTER.

VOL. L.] OCTOBER, 1896. [No. 10.

### PROCEEDINGS.

### Proceedings of the Meeting of the Michigan State Dental Association, Grand Rapids, Mich., June 10, 11 and 12, 1896.

AFTERNOON SESSION.

The meeting was called to order by President House, and was opened with prayer by Dr. Morgan.

THE PRESIDENT: I regret that we have not a larger number present at the opening of this meeting. We generally have a fair representation at the beginning, but I suppose they will come later.

We will have an address of welcome from Mayor L. C. Stowe, and I take pleasure in introducing to you the Mayor of our city, the Honorable L. C. Stowe.

MAYOR STOWE: *Mr. President and Gentlemen of the Michigan State Dental Ass'n:* It affords me great pleasure, in behalf of the citizens of our city, to extend to you our cordial greeting and a most hearty welcome. You represent a profession that commands the respect of all classes of people, and while you all seem to have a kind, genial, sympathetic look in your faces, yet, I believe you are the cause of more intense pain than any class of people on the face of this globe. Notwithstanding all this, we are glad to have you with us.

The science of dentistry has made rapid advances—probably as much so as any of the other sciences. Mr. President, it has evolved from the old twisting, crushing, jaw-breaking turn-key to the up-to-date "teeth pulled without pain," as I am informed by the advertisements I see in our daily papers.

Gentlemen, I am glad to see your society so well represented at this, the first, meeting, and I trust your convention will prove to be a profitable and an enjoyable one. I do not wish, gentlemen, to consume your time, which is valuable, and I will again bid you a hearty welcome.

The President: I had expected that Dr. Taft would be here to respond to this address of welcome by Mayor Stowe. We received a dispatch that he missed his train. I can not tell whether it was intentional, Mr. Mayor, but we will have Professor Hoff respond to this address of welcome.

Prof. Hoff: *Mr. Mayor, President and Gentlemen of the Society:* I feel wholly inadequate to make any reasonable answer to the hearty welcome that we have just received. I hope, Mr. Mayor, that you will have no difficulty in policing this body; it certainly is not a very large one. I presume you have a larger police force in the city than we number here, but there will be more of us later on, and, perhaps, when you come to handle us you will think there are a great many more of us than there seems to be. We are very grateful to you for your kind invitation and the hospitality you have extended to us; we appreciate it and the beautiful city to which you have welcomed us—and this magnificent weather is certainly all that we can desire, and this beautiful place of meeting, it seems to me, is a most auspicious opening of our meeting.

While many of our members are not here, we have a representative number; we never can hope to get together all of our profession in any of our meetings, but we always get together a representative body. You see before you the representative dentists of the State; this body represents the dental profession in Michigan, so far as it is possible to represent it in an organized form, and we hope in this association to do whatever may be done by the profession in an organization, and not only bring the profession together, bring the individuals together in an association which shall accomplish more than we can hope to do in any individual capacity, but we hope by united effort to accomplish a very great deal toward elevating our profession to that plane which will compel a recognition from the public of its value and its necessity. We realize that dentistry is a noble calling; we feel honored to be members of it. I have no doubt, if you were to canvas this body, or any body of dentists, you would find that a very large majority of them were satisfied with their calling in life. A great many think dentistry is a small and circumscribed occupation and that a man who enters it has a narrow view of life, but dental science, dental art, has opened up so much, that those who are best acquainted with it are surprised, at times, in looking at the opportunities we have, and the broadness of it; it is so broad that one who does not know of its capacity can have no very adequate idea of it. And so I, for one, feel that we have a right to be recognized by the citizens of our State; we represent the organization of the best element in our profession in this State and we claim the recognition of the best element in society and of the State, and all organized society everywhere, and we hope to merit that recognition by doing whatever we can for the benefit of the public in general; we are organized for that purpose; we are not a trust, that is, we are not organized for a selfish purpose or personal or professional aggrandizement; we are organized that we may stimulate each other to better work, to better effort, and by doing better work and putting forth better efforts we hope to serve the public to much better advantage, and it is with that in view that we are here to-day—not that we may personally get anything that shall be of any personal benefit to us, but that whatever we may gather up, whatever we may get here shall look to the ultimate good of the public and for the benefit of the public at large, and on these grounds we claim your recognition, and we accept your hospitality and shall cherish it and use it as we may have opportunity. We thank you for it.

It was moved and seconded that the calling of the roll be dispensed with until the evening session, which motion was carried.

The report of the Board of Directors was then read by the secretary, which report was accepted.

Dr. J. A. Robinson was called upon to read his paper entitled "Then and Now."

(See August No. Dental Register, page 396.)

The President: Before we take up the next paper, we have with us to-day a gentleman, Dr. Adams, of Toronto, a member of the profssion in Ontario, and a delegate to the National Association of Organized Charities. He has been interested, for some years, in the care of the teeth of the worthy poor, and to such an extent that he has been able to have established in the city of Toronto a dental health inspector, and if I understood him aright, a dental hospital where the worthy poor can have their teeth taken care of, and if it is the pleasure of this meeting I would be pleased to have Dr. Adams address us.

On motion of Dr. Hoff, Dr. Adams was requested to address the association, and responded as follows:

Mr. President and Fellow-Workers: It affords me much pleasure to meet with you here to-day. I did not expect, when I came to your city, to have such a pleasure, but I met your president and he kindly asked me to speak to you for a few minutes on the work in which I am especially engaged.

SCHOOL-CHILDREN'S TEETH—THEIR UNIVERSALLY UNHEALTHY AND NEGLECTED CONDITION.

In the closing years of this nineteenth century I am sure the public do not wish to be kept in ignorance of any danger that threatens the health and prospects of the rising generation.

I, therefore, wish to lay before you some facts which I have gathered from experience and personal observation and investigation in reference to the sad condition of children's permanent teeth of the present day. To my mind there is no subject that should be of more interest to you than this, whether viewed from a *parental*, *sanitary*, *educational*, *scientific* or *humane* standpoint; it is one that is thrilling with interest.

On this subject very little has been written, but the universally-unhealthy and neglected condition of children's teeth is such that silence on my part, knowing their condition as I do, would be criminal, for very few parents are aware of the wholesale sacrifice of their children's permanent teeth; as they suppose that the teeth that are aching are their first teeth. But if they could see their condition, as I have, they would be alarmed and would, methinks, do something to prevent it.

For the past twenty-four years, in addition to caring for the teeth of the children in my regular practice, I have, with the aid of assistants, carried on Dental Hospital work among the children of the poor of Toronto, filling the teeth free for as many such children as I could gather in. Beside this, I have examined the teeth of large numbers of children in the public schools of the leading cities of Canada, and of some of the American city schools, including the largest German school on the continent. I have, furthermore, examined the teeth of the Indian tribes on the Georgian Bay, and of hundreds of children just out from England, besides a number from Syria, Russia and Japan. The examination of so many thousands of children, comprising those of different nationalities, has given me an exceptional opportunity of noting the condition and the change that is taking place in their teeth. I shall not here touch upon the cause, but shall confine myself to the condition, as I find it, and to the only present practical remedy.

I find that children's teeth decay at a much earlier period than they did formerly, and that the quality of the teeth is so much inferior, that unless they are filled as soon as they begin to decay, and while the cavities are small, they are soon past all hope of being saved. I am speaking of the permanent teeth—not only of the sixth-year molars, but also of the twelfth-year molars, bicuspids and superior incisors, which now very frequently begin to decay within a year or two after being erupted. I have examined the teeth of a large number of adults from fifty to seventy years of age who, like myself, have first-class sets of teeth, far better now than 95 percent of the children of to-day. In every city I visited I found the teeth of the children in the same neglected condition. Though almost every child had teeth requiring to be filled, there were not 5 percent who had any filling done. I found only one child whose teeth had been filled at the right time; the rest had been neglected until they were very far gone, and were hardly worth being filled, showing that their parents were not systematic in attending to them, but only did so when the children had suffered very much. I did not find, in the schools, 3 percent of the children with as good teeth as I have at fifty-seven years of age.

In every kindergarten I examined I found many of the children with from two to four of their permanent teeth decayed, while mine, that I got at their age, are perfectly sound. I can not, by words, picture the exceedingly unhealthy condition of the teeth and mouths of a large percentage of the children in the various schools I examined. About from 1 to 5 percent of the children had sound sets of teeth; 15 percent had teeth fairly good, but some of them requiring to be filled; about 50 percent had many teeth decayed, some of which were so very badly decayed as to make it difficult to save them, as they would require to be treated for days or weeks, and that heathenish operation performed—the destroying of the nerve-pulp, an operation that should never be performed on a child—and yet, what can the dentist do when the parents bring their children in such a neglected condition? He has either to destroy the nerve or extract the permanent tooth, which operation is a still more cruel and barbarous thing to do. About 30 percent of the children I found in a still more neglected condition; the teeth and mouths of many of them were so disgusting that no dentist would think of working for them until their mouths had been disinfected. Many of the children under twelve years of age had from eight to twenty permanent teeth in various stages of decay; large numbers of them were dead teeth, mere shells, filled with decomposing food; other teeth were abscessed and the gums covered with vile, disgusting pus, which in many cases was very copious.

How shall I describe the furred condition of the tongue, and the foul gases emitted from the mouths of such children, which were veritable hot-beds for every species of bacteria, having all the elements necessary for germination—heat, moisture, decomposing food and teeth, together with the foul gases arising from them and the stomach! What better conditions could bacteria have? Our health authorities are very careful about having all bones and refuse removed from yards to prevent the air from being polluted ; and yet school-children by the tens of thousands are compelled every day to bring their vile, dead, rotten *bones* to school to contaminate the air of the overcrowded rooms and spread disease among the children whose parents have been careful about their teeth. When sickness breaks out in the school (and it is often doing so) the health officer, at great expense, searches the buildings, drains and closets, to find the cause, not suspecting that it is often in the children themselves who have been weakened by slow poison.

I consider it a crime to compel teachers and children who take good care of their teeth to sit in the same room with such children. Remember, many of these were not poor children whose parents were not able to get their teeth filled, but children whose parents were in comfortable circumstances and were both willing and able to care for them, but, unfortunately, they were not aware that their children's teeth were in such a condition, but supposed that the teeth that were decaying were their first teeth and that Nature was helping to get rid of them by decay, in order that new teeth might take their place.

In every school I examined I found children whose permanent teeth had forced the roots of the deciduous teeth out through the alveolar process, and the rough, jagged points had lacerated and worn away the cheeks and lips, making a hole, in many cases, large enough to hold a walnut. Cancers often result (though not in children) from such laceration! I found many of the children who, for months, had not been able to masticate solid food, and their pinched, half-starved faces told how they were being injured.

Mr. Levi Clark, Principal of one of our schools, said, at a public meeting in the interests of the children, that the result of the examination of the children's teeth in his school was a revelation to him, and that he could not see how it was possible for the children to attend school at all, with their teeth in such a shocking condition. But he said they were so ambitious to get their certificates that they would continue at their studies even while suffering great pain, and would come to school with their faces swollen and covered with tears, but that at last they were compelled to go home.

The examination of 25,000 city school children, some in Canada and some in the United States, shows that one-half of them have suffered so much from abscessed teeth at night they could not sleep; one-fourth of the children were not able to attend school, some for days and many for weeks; and that out of these 25,000 children only 2,200 had teeth filled this year, though most of them belonged to the well-to-do class of our cities. There are more than 100,000 permanent teeth in the mouths of the school-children in Toronto alone that are going to destruction without any effort on the part of their parents to save them. Who can estimate the unnecessary loss sustained or the suffering endured by these children, and the millions of others on this continent who are in like condition? 'Tis a sum that can not be computed, for the effects will not end with the death of the children, for those who live long enough to marry will hand it down to their children, and the world will be populated with nervous dyspeptics!

I shall now point out some of the ways by which the children whose teeth have been neglected are injured, and then show how the bad condition of their teeth affects the health of the teachers and of the other children in the school whose teeth are kept in good condition. First, those neglected children suffer excruciating pain with toothache and neuralgia, often for weeks at a time, so that they can not eat, sleep, or study, and consequently become nervous and irritable. This prevents them from succeeding with their studies, and they soon fall behind their class and become discouraged, thus interfering very much with their education. In the second place, these children (not being able to properly masticate their food, which is consequently not thoroughly mixed with the saliva) bolt down their food in coarse chunks which irritate the stomach and bowels, causing indigestion, dyspepsia and diarrhœa. An eminent English doctor, writing on this subject, says that he considers that the rapid increase of intestinal troubles among the children in England, so often ending in death, is attributable to the unhealthy condition of their teeth.

In the June number of an English quarterly magazine, published by C. Ash & Son, I found an interesting paper on the decay of the teeth in the national schools of Germany, written by Dr. C. Rose, of Freiburg, Baden, and read at a meeting held in Vienna, in which he shows that the children's teeth there also are deteriorating very fast, and that very little attention is given to their preservation. In one district, out of 6,300 school-children examined by him 98 percent had decayed permanent teeth, and yet out of that number only twenty-seven children had teeth filled. He further says that in various places which, during the last few years, had been heavily scourged with diphtheria epidemics, it struck him that the children, in their thirteenth and fourteenth years, possessed exceptionally good teeth in comparison with those of more tender years. This fact, he says, can only be explained in this way: That the children with bad teeth had, to a greater extent, succumbed to severe forms of diphtheria, inflammation of the lungs, and other infectious diseases. He says that Miller and others, it is known, have shown that most of the disease-bearing fungi appear, at times, in the unclean mouths of healthy people.

In his opinion, during an epidemic, the diphtheretic fungus will penetrate, for a time, to the mouths of nearly all the children in a school. He says: "In a clean, well-cared-for mouth, it does not find favorable conditions for its development, but grows vigorously in and near the roots of the teeth of a neglected mouth. The most effective sanitary measure in the treatment of diphtheria, during the disease and before, consists of the frequent thorough cleansing of the mouth and throat. A mouth with good teeth and tense gums can naturally be cleansed by rinsing, and much more easily than can one with diseased roots and loose gums. The most important preventive sanitary measure on the appearance of any epidemic is the most careful attention to the mouth."

His investigations reached the figures of over 13,000 school-children. "The results of these investigations," he says, "should be sufficient to convince the German governments of the necessity of dental hygienic measures in the national schools. This is a duty the governments can not shirk."

I am pleased to be able to give you these extracts from his paper, as they so fully correspond with my own experience in this country, and add greater interest to this subject.

I have now given you the condition of the children's teeth, not only on this continent, but elsewhere, showing conclusively that this deterioration of children's teeth is universal. I leave it to you to judge what effect this condition of the children's teeth has on the health of the schools and the poor, and ask, Is it not time that something be done to prevent this fast increasing evil, instead of trying to relieve pain and heal the disease? The true sanitarian by anticipation goes before it saying "Halt, you can not enter here!" In England they are waking up in earnest and in many of their training schools are not only examining the teeth of the children, but are employing dentists to fill them. They will not receive applicants to either the civil or postal service who have decayed teeth, and are very strict in their examinations, for they say if their teeth are bad, their health will break down, and they will be placed too soon on the pension list. They have also established a dental hospital where the poor can have their teeth cared for.

It is one thing to diagnose a disease; the next is to provide and apply a remedy. The only remedy at present is to fill the teeth as soon as they commence to decay, long before the nerve pulp has become exposed or the tooth has ached and while it can be done at one sitting. But just here comes the great difficulty, which doubly increases the seriousness of the evil. The parents are not aware that their children's teeth that are aching are *permanent* teeth, until they take them to their dentist to have their teeth extracted, and then to their surprise find that they are permanent teeth, and past all hope of being saved. There is not more than one parent in a thousand who knows the difference between the temporary and permanent teeth. For many years I have found it difficult to get the children in the charitable homes to come to me in time to save their teeth. They would only come when their teeth were aching and often they would be past saving, or if they could be saved it would take as much of my assistant's time to fill one bad tooth as it would to fill eight or ten if filled at the right time. So I adopted the plan of going to these schools twice a year to examine their teeth, and those whose teeth needed filling were then sent to me in the dental hospital.

This plan has been a great success in these schools, for now we never have any bad teeth to fill, nor any permanent teeth to extract, and the mouths of the children are clean and healthy. The contrast between the condition of the teeth of the children in these schools and those of the well-to-do schools of our city where this system has not been adopted, is sufficient, I am sure, to convince every fair-minded person that this system is not a fad, but an absolute necessity. Let me ask, why should not all parents in the cities and towns on this continent be informed of the condition of their children's teeth, in time to save them, and thus prevent this suffering?

I have given this subject years of careful study, and have failed to find any other remedy equal to this. I would therefore suggest, in the interest of our school-children, that in all our cities and towns a dental health inspector should be appointed whose duty it would be to examine the teeth of all the children, twice a year (except those children who brought a certificate from their family dentist saying their teeth were being attended to), and fill out reports to be taken home by the children to their parents stating the condition of their teeth, and advising them to send their children to their family dentist before their teeth were past saving. As there are large numbers of parents who can not pay much, and some who can not pay anything for having their children's teeth attended to, it will be necessary to establish a dental hospital in each of the towns and cities where such children can have their teeth filled and cared for at a nominal fee, simply enough charged to pay expenses. The whole of this work can be carried on with little or no expense to the city or State, as the dental health inspector can fill both office of inspector and also that of superintendent of the dental hospital; he can spare one or two hours every morning to make the examinations, or sufficient time to go over the schools twice a year. This would be better than completing the examinations in a few weeks, for it would give the dentists and hospitals time to do the filling without being crowded, or the danger that some of the children would be forgotten who could not be attended to at once.

The examination will not interfere with the work of the school as it will not take more than thirty minutes to examine all the children in the room. I have examined one hundred and fifty children in an hour, and one hundred can be examined with ease. A large number of the school-children will go to their family dentists to be examined, which will lessen the work of the inspector and the number that will do so will yearly increase as they become educated, and thus all the school children, rich and poor alike, will be systematically examined twice a year. I am satisfied that by this plan the expense to the city, if any, will be very small, while the advantage to the rising generation will be incalculable, and will put an end to the present barbarous practice of wrenching out from the delicate jaws of so many children, the permanent teeth that God has given them to masticate their food and to beautify their features.

Sometime ago I sent out circulars to the leading cities in Canada and the United States, asking what these cities were doing for the preservation of the teeth of the children of the poor. The replies so far received, are that there is not anything being done except in the few cities where they have an infirmary connected with a dental college, and these infirmaries are not open all day, nor all the year ; so that even in these cities there is not much work done for the children of the poor. In all of the other cities they are utterly neglected.

The dental hospital, that I have provided for the children of the poor of Toronto, is the only one of the kind, at present, on this continent. The poor have no idea of the value of their teeth and we are responsible for their education on this subject. In their interests and in the interests of their children after them, as well as in the interests of the school-children that have to sit day after day with them, they must be cared for and if necessary be compelled to have their teeth filled.

I shall just here point out the difference between a dental and a general hospital. The former, not having any resident or fever patients, will therefore not require so large or expensive buildings; nor to be placed at some distance from the resident portions of the city. It may be carried on in a rented building if need be, and in such part of the city where rent is not so high, and where it will be the most convenient to that class of the citizens whom it is intended to benefit. Again, it is different from the general hospital, inasmuch as it is not open to any person who is able to go to a dentist and pay the usual fee; for while the dental hospital should be self-sustained and not a burden on the taxpayer, it should not be made a money-making institution, competing with those persons who have to make their living by the practice of dentistry. It should only be open to two classes of citizens: (1) To those persons who, though able to pay something for the work they require, yet are not able to pay what a dentist having to keep up an expensive office, would necessarily have to charge. (2) To those persons, such as I have just described, who are not able to pay anything.

I shall not point out another difference between a dental and a general hospital, and which greatly helps in carrying out the work of the former. In the general hospital it cannot be known beforehand who the patients will be or what their ailments are, and therefore appointments cannot be made for them to come in when it will be most convenient for the hospital staff to attend to them; but in the dental hospital, as far as these children of whom I have been speaking and who are not able to pay are concerned, it is quite different. We know that they require attending to every six months, no guess-work, and therefore if we have their address, we can make appointments for them, just when they should be cared for, and when we can attend to them; thus, we can have the work of the hospital going on, as far as this class is concerned, in a steady, systematic manner—not a large number, more than can be attended to in one day, coming in; and then perhaps the chairs standing empty for days as has been my trying experience, with the additional dissatisfaction of knowing that the work we have done for them, at so much expense and trouble, will be largely lost for our want of being able to keep track of these patients, as many of them do not return for a year or two, until the teeth we have filled have been destroyed by others that have in the meantime been neglected. The suffering of such children is terrible, as I know from what I have seen among them here in Toronto during the past twenty-four years —suffering such that no tongue can fully describe?

I have also given you some idea of the suffering endured and the loss sustained by the millions of poor children on this continent caused by the neglected condition of their teeth. Though we call this, not only a civilized, but a Christian land, yet the Christian, humane, and well-to-do people in it have, in no city on this continent provided a dental hospital for the preservation of the teeth of these poor children, nor have they tried to prevent this untold suffering. I do not know how it looks to others, but to me, having seen so much of this suffering, it seems an awful thing, that in the closing years of this nineteenth century, in which we boast of such great educational, sanitary and humane reforms, that millions of little human beings who had no choice whatever in coming into this world of suffering, should, for lack of that Christ-like care which Christians are expected to evince, be compelled, after months of suffering, to be subjected to that cruel and barbarous operation of having their permanent teeth, that God, their Heavenly Father has so kindly given them to masticate their food and to beautify their features, wrenched out of their delicate jaws.

We talk about the ignorance and cruelty of the heathen ; and and yet on this continent, claiming to be highly civilized, the cry of millions of suffering, fatherless and neglected poor children is going up to Heaven, night and day, one unceasing wail, while for want of knowing better, their parents take them "like lambs to the slaughter." Let me ask: In what heathen country can you find so many parents offering up to their gods such a costly or cruel sacrifice as do the parents on this continent, in the sacrifice of their children's teeth, health and beauty, on the altars of this hideous god, ignorance, whose constant cry is,—"Give!"

" Give!" and yet is never satisfied, unless his altar is overflowing with the blood of these human victims?

To sum up the whole subject and to save time, I shall just state some self-evident facts which it will not be necessary to bring evidence to prove:

1. Children whose teeth are neglected, in the way I have described, cannot eat, sleep, study, or play the same as those children whose teeth are keep in first-class condition; therefore they become nervous and irritable.

2. If their food is not properly masticated and mixed with the saliva, but bolted down in coarse chunks, it will not be well digested and therefore not assimilated, the result being that though they have eaten the necessary amount of food, yet their systems are not built up.

3. The coarse food "bolted down," irritates the stomach and bowels, causing indigestion or dyspepsia and intestinal troubles. An eminent English doctor says that he considers that rapid increase of intestinal troubles among the children in England, which so often ends in death, is directly attributable to the unhealthy condition of their teeth.

4. Such children are a menace to the other children in the schools, because their systems being so susceptible to disease, they, unlike healthy children, cannot pass an open sewer without inhaling some virulent microbe germs, which find in their decayed teeth, inflamed gums, and generally run-down systems, a hotbed where they can soon multiply by the millions; and then becoming strong by numbers, they are able to attack the more healthy children in the school, and thus disease spreads, until the schools have to be closed.

5. Children whose teeth have been neglected, have not the same chance to recover from disease as those whose teeth are kept in a good, healthy condition; not only because their systems are weaker to start with, but also because of the extra suffering they have to endure while sick, caused by toothache and neuralgia, which are sure to trouble them more when confined to bed, thus the temperature is greatly increased and the disease more complicated.

6. If the disease is contagious, a dentist will hardly care to venture into an isolated hospital to relieve the children lest he should convey the disease to his juvenile patients.

I shall now give you a few reasons why we, as a Christian people, cannot neglect the children of the poor:

1. Because we profess to believe God's Word which says, "He is the husband of the widow, and the father of the fatherless and that the poor are His peculiar care, and that whoso touches them touches the apple of his eye."

Moreover:

"Whoso stopped his ears at the cry of the poor, he also shall cry himself but shall not be heard." Pro., 21 ch., 13 ver. We say we believe in the Fatherhood of God, and the Brotherhood of Man; therefore we are compelled to show by our actions that we are consistent.

2. Because these children have sufferings enough to endure through life, without being compelled to bear any that can be foreseen and prevented.

3. Because their suffering from toothache and neuralgia which they endure so much at night is much greater than what is borne by the well-to-do, for they are compelled to lie in cold, comfortless homes which they cannot afford to keep warm at night which is the time when they suffer the most from neuralgia.

Looking at this subject from a selfish point of view, we cannot afford to neglect the children of the poor:

1. Because we do not know how soon we may have to change places with them, as riches often "take wings and fly away."

2. Because we cannot afford to let our children sit in the same room, side by side, with these neglected children and inhale the vile gases, constantly emanating from them, caused by their rotten teeth and the decomposing, undigested food in their stomachs which, passing from them, pollutes the air of the room, and is heated up and breathed over and over by our children, and it is always getting viler as the hours pass by.

3. Because we cannot afford to let our children drink out of the same cup these children have fouled with the poisonous pus which is constantly exuding from the gums around their abscessed teeth and roots.

4. Neither can we allow our innocent little children to be exposed to the danger of chewing gum that has been crunched into the disgusting teeth of some of their playmates.

5. These children will soon be our employes, and we can not afford to have a nurse with our children who has had bad teeth, tasting their food and cooling it by blowing her vile breath over it. They may, perchance, be our servants, preparing our food, putting the spoons that they have just taken out of their mouths back again and again into the fruit and other dishes which they are preparing for us. Not a very pleasant thought for any person who has seen the condition of their teeth as I have.

6. These persons will not be as healthy as they would have been if they had been cared for when young, and, therefore, can not give us the same return for our money as they should have.

When, by school-inspection and dental-hospital provision, 90 percent of this loss and suffering can be prevented, and that without increasing the taxes of the citizens, I am sure that every person interested in the rising generation will be anxious to have this much-needed reform begun, in every city on this continent.

Now, in conclusion, I might say that during the past years I have been unable to reach the children of the poor, who were so scattered through the city, in time to save their teeth, so that though the expense of the hospital was going on, the work for which I was spending my time and money was not being accomplished.

I have now been forced to adopt the following plan, and one which, I think, will have to be adopted by every dental hospital that desires to reach the children of the poor in time to save their teeth. It is this: To advertise for the name and address of all poor women who have children depending on them for support. Having thus got the address of such, I send a visitor to see them. If they are not able to pay anything for dental services, but are willing to have their children's teeth systematically cared for twice a year at whatever time I may appoint for them, I then place their names on the free list and they will be cared for at my expense. All other children coming into the hospital will be charged a small fee, according to their circumstances.

DR. FIELD, of Detroit: I regret that this paper has been read at this time when we have so few to hear it. It is certainly a very interesting and exhaustive paper, and one that appeals to me most heartily for its earnestness and for the understanding manner in which it has been written.

In my position as teacher, in the schools of Detroit, since I have been there, I have had more of an opportunity of seeing just such cases as the gentleman speaks of in his paper that we have listened to, and I understand thoroughly and can appreciate what he speaks of. I have been approached on this matter myself, and I am not certain but that I received the paper that this gentleman says he sent through Canada and the States, appealing to the dentists to take some part in it, but there is somewhat a matter of delicacy in dentists taking a position of that kind in the schools in the States, on account of so many of the families having their own private dentists. The question would be whether they would not feel that they were infringing upon something with which they had no business. I think it would be perfectly right, and there could be no wrong in it, yet, at the same time, a man might have some delicacy about doing it, but, at the same time, it meets with my hearty approval, and I think it is an excellent paper, and I am only sorry it has been read before so few as we have here to-day. I move a hearty vote of thanks to Dr. Adams for presenting it to us.

DR. J. C. PARKER, of Grand Rapids: I wish to add to the remarks of Dr. Field my hearty concurrence in what he says, and I think that nothing could be brought more opportunely before this association than this paper.

Dr. Field speaks of the delicacy we feel about it. I hardly think that would matter because we have no feeling of delicacy in regard to the compulsory examination of children in regard to vaccination, and as soon as this matter was thoroughly settled it would become a matter of course that the children in our schools would be examined twice a year as to the condition of their teeth just as they are for vaccination. I would support the the motion of Dr. Field.

The motion to tender Dr. Adams a vote of thanks was unanimously carried.

Dr. Adams: I thank you, gentlemen, for your vote of thanks, and would like to say just a few words if you will give me a minute or two more. I think, probably, you did not just understand what I meant about the compulsory examination. What I proposed was this: not that all children should be examined systematically twice a year, but they would not necessarily be examined in the school at all; they could all go to their family dentist and there be examined; that the inspector, before going to a school would send word to the principal a couple of weeks beforehand saying that he would be there to examine the children's teeth in a couple of weeks, and the principal could then notify the children and all that wished to go to their family dentists could go; it would be compulsory, but the better class could go to their family dentists and the poor would be inspected, and all the well-to-do, of course, would go to their family dentists, so that all the inspector would have to do would be to examine the poor who would neglect to do so; so that it would not interfere with the rights of the well-to-do, or conflict with them at all.

